# Gender-Related Trends in Publication Authorship: A 10-Year Analysis of a Brazilian Surgical Journal

**DOI:** 10.7759/cureus.18993

**Published:** 2021-10-23

**Authors:** Sarah B Motter, Gabriela R Brandão, Júlia Iaroseski, Amanda V Alves, Ana Luíza K Konopka, Candida M de Assis Brasil, Gabriela S Silva, Joana L Spadoa, Rosilene J Reis

**Affiliations:** 1 Medicine, Federal University of Health Sciences of Porto Alegre, Porto Alegre, BRA; 2 Department of Gynecology and Obstetrics, Federal University of Health Sciences of Porto Alegre, Porto Alegre, BRA

**Keywords:** sex inequality, female representation, surgery, authorship, surgical literature, brazil

## Abstract

There is a gender gap in the representation of women in the authorship of surgical literature worldwide. In Brazil, data on the gender distribution of the authorship of articles are scarce; and hence, there is a lack of awareness about the contemporary situation of women surgeons within the academic surgery in the country. In light of this, we conducted this study with an aim to describe and evaluate the authorship trends in a Brazilian surgical journal over a period of 10 years (2010-2019). We included 4,301 authors from 792 articles extracted from 60 editions of this journal. We analyzed the female representation as authors in general, first and last authors, and the female surgeons' representation as first and last authors for 568 original articles. We found that, in general, women represented 27.8% of all authors. Regarding original articles, women surgeons represented 8.4% and 6.1% of first and last authors, respectively. The linear regression analysis demonstrated that there was an increase over the years in women authorship. However, despite this increase over the years, a gender gap still persists in terms of women's representation as authors in the Brazilian surgical literature.

## Introduction

Even though women were historically considered "owners of medical expertise", owing to their roles as midwives and healers, they have been constantly fighting to establish their place in the field of modern medicine [1]. Today, unfortunately, there is a persistent gender disparity in the medical community. This scenario of gender inequality is particularly evident in certain areas of medicine, such as academic research, a field where women are still very underrepresented [2-5]. Publishing in medical journals is an important measure of academic productivity and an essential communication practice within the medical community. In the last few years, women's authorship representation has been increasing in medical journals; however, when compared to men, their proportion is still low: about 15-25% [2,6]. Surgery represents one of the medical specialties where these contrasts are more obvious and striking. According to a study by Jagsi et al. [2], in 1970, only 2.3% and 0.7% of articles in the Annals of Surgery had women as first authors and senior authors, respectively; in 2004, these numbers increased to 16.7% and 6.7%, respectively. In this study, the authors also identified the lower rate of increase in women's surgical academic participation over the analyzed period (1990-2005) compared to overall medical specialties by considering metrics such as women's participation as first authors in the journal Annals of Internal Medicine (4.7% to 31.5%) compared to that in the publication Annals of Surgery (2.3 to 16.7%) [2]. Thus, it is evident that there is a gender gap in the representation of women as authors, especially in the surgical literature, with women rarely publishing as senior authors (4-6% of articles) [2,7]. Regarding the scenario in Brazil, aspects related to gender distribution in article authorship have been scarcely explored, and hence there is little awareness about the contemporary situation of woman surgeons within academic surgery in the country. Hence, more studies that focus on the inconclusive aspects of this gender gap are required. Therefore, given that authorship patterns may shed light on women’s participation in the surgical academic field, it is important to analyze if the lack of representation of women in academic publications, as seen globally, also applies to Brazil. Thus, this study aimed to investigate the aspects related to gender representation in terms of article authorship in a surgical journal in Brazil, with a special focus on authors who are surgeons and females.

## Materials and methods

Data source

For the purpose of this study, we chose the journal of the main Brazilian surgical association, which represents the largest organization of surgeons in Latin America. The journal of this association is indexed on Latindex, Lilacs, Scopus, Medline/PubMed, Directory of Open Access Journals (DOAJ), and Free Medical Journals. We chose this publication because it is the most representative of the Brazilian surgical academic scenario. The name of the journal analyzed is not revealed due to ethical reasons concerning research standards in Brazil. 

For our analysis, authorship data from 60 editions of this surgical journal published in a 10-year period between 2010 and 2019, amounting to 792 articles, were collected. Elements of this data included the name of the published article, article category (original article, case report, technical note, evidence-based medicine, teaching, bioethics, and letter to the editor), date of publication, a complete list of authors of each article, and affiliation of the first and last authors of original articles.

Classification of authors and sampling criteria

A total of 4,301 authors were counted initially, which were further classified into first, middle, or last authors according to the article's authorship order. The authors' gender was classified as female, male, or unknown according to our knowledge about the gender traditionally associated with their names in Brazil (e.g., Maria as female, and João as male). The gender was determined by two authors who were blinded to the details of the study, and in case of a conflict, a third author would conduct an active search of public profiles in an attempt to determine the gender of the name in dispute. In cases where it was impossible to determine the gender, the author was classified as unknown gender (42 in total), which were counted in the category of total authors but excluded from the other analyses. To determine whether the first or last author was a surgeon or not, we considered the affiliation revealed by the author in the original publication; thus, our classification was based on the professional's educational status at the time of publication of the article. If the affiliation was explicitly provided as "surgeon", we considered him/her as a surgeon. If another specialty/profession was explicitly provided, or if it was a resident who had not yet completed the general surgery residency, we considered him/her as a non-surgeon. In Brazil, designations related to medical specialties, e.g., surgeons, are acquired through the completion of the medical residency program or by the evaluation of a specialized society recognized by the Federal Council of Medicine. In cases where this information was not explicit (e.g., professor of surgery, doctor of the surgery team, etc.), we conducted an active web search, using platforms such as the Federal Council of Medicine [8], Curriculum Lattes [9], and public profiles to determine if the author was a surgeon or not at the time of the concerned article's publication. Authors with foreign affiliations were excluded from the analysis since our objective was to assess a Brazilian trend in authorship in the surgical journal, which would contrast with international trends. In total, as mentioned above, 4,301 authors were initially included. However, for the general analysis, articles of the following nature were excluded: those with institutional authorship instead of individuals (e.g. “national surgical committee''), authors with explicitly foreign affiliation, and authors whose gender could not be identified. Therefore, the final analysis was based on a sample of 4,225 authors. To evaluate the authors as surgeons and non-surgeons, considering the kind of active search and considering the leadership roles in the manuscript process, we included only the first and last authors of original articles. We excluded in this analysis 224 articles that did not represent primary research articles (e.g., comments, letters, editorials). Thus, 1,136 authors were classified as surgeons or non-surgeons from 568 original articles.

Data analysis

All data were recorded and initially stored in Excel spreadsheets (Microsoft Office Excel 2003; Microsoft Corporation, Redmond, WA), and analyzed using the SPSS Statistics software version 25 (IBM, Armonk, NY). Binomial statistical tests were used to test the difference in proportions between men and women among authors; a chi-square test was used to test the cross-difference between men and women as first and last authors; and linear regression analysis was employed to test the alternative hypothesis of female representation increase in general, as first authors, as last authors, and as surgeon first authors and surgeon last authors of original articles.

## Results

Prevalence of female authors

For the 792 articles analyzed in total, we found a total of 4,225 authors; of them, 3,050 (72.2%) were men and 1,175 (27.8%) were women. There were 75.0% men and 25.0% women in the position of the first author; and 78.2% men and 21.8% women in the position of the last author. Regarding original articles (n=568), we found a total of 568 first and last authors, with 72.7% and 78.0% of them respectively male, and 27.3% and 22.0% of them respectively female. All of these differences in proportion between women and men were statistically significant (p<0.001). (Table 1).

**Table 1:** Frequencies and analysis of the difference in proportions between male and female authors *Binomial test for differences in proportions

			Male, n (%)	Female, n (%)	Total, n (%)	P-value*
All articles (n=792)						
	All authors		3,050 (72.2)	1,175 (27.8)	4,225 (100)	<0.001
		First authors	594 (75.0)	298 (25.0)	792 (100)	<0.001
		Last authors	619 (78.2)	173 (21.8)	792 (100)	<0.001
Original articles (n=568)						
	First authors		413 (72.7)	155 (27.3)	568 (100)	<0.001
		Surgeon	298 (52.5)	48 (8.4)	346 (60.9)	<0.001
		Non-surgeon	115 (20.2)	107 (18.8)	222 (39.1)	<0.001
	Last authors		443 (78.0)	125 (22.0)	568 (100)	<0.001
		Surgeon	303 (53.3)	34 (6.1)	337 (59.4)	<0.001
		Non-surgeon	140 (24.6)	91 (16)	231 (40.6)	<0.001

Prevalence of female surgeon authors

Among the first and last authors of original articles, we observed that 60.9% and 59.4% were surgeons respectively. Of all the 568 first authors of original articles, 52.5% were surgeons and men, and 8.4% were surgeons and women. Of all the 568 last authors of original articles, 53.3% were surgeons and men, and 6.1% were surgeons and women. There was also a statistically significant proportional difference between women and men in this analysis (p<0.001). (Table 1).

Representation of female authorship over the years

**Figure 1:**
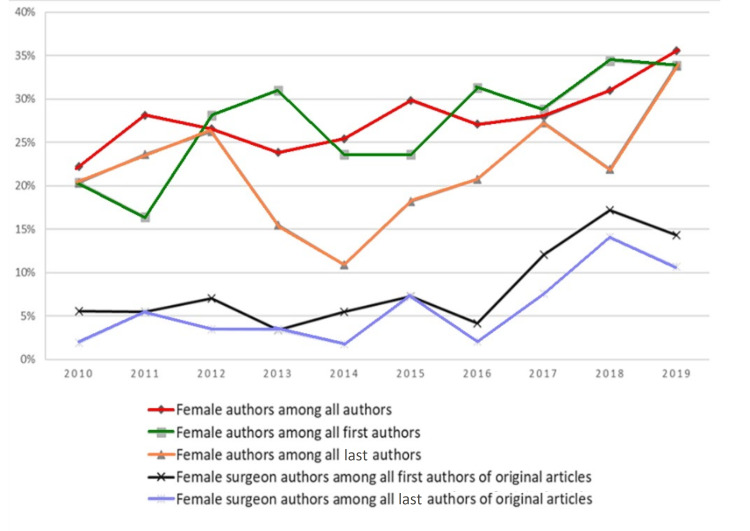
Gender trends related to female authors among all samples and original articles (2010-2019)

Among all authors in the 10 years analyzed, there was a significant increase in the number of women authors (p<0.01) (Figure 1). In 2010, women constituted 22.2% of the authors, and, in 2019, they represented 35.6% (Table 2).

Regarding the positions of the first and last authors, there was an increase in women among the first authors; however, that was not the case with the last authors. The general proportion of female first authors greatly varied, constituting 20.4% in 2010, which dropped to 16.4% in the following year, and reached 33.9% in 2019, showing significant linear growth (p<0.01). On the other hand, the general proportion of the female last authors did not show a significant linear increase (p=0.253), with the years 2013 and 2014 reporting a decrease (Table 2).

**Table 2:** Representation of female authors among all samples and original articles (2010-2019) *Linear regression analysis for trend

			2010	2011	2012	2013	2014	2015	2016	2017	2018	2019	P-value*
			Proportion (%)										
Total sample													
	Female authors												
		Among all authors	22.2	28.1	26.6	23.9	25.4	29.8	27.1	28.0	31.0	35.6	0.007
		Among all first authors	20.4	16.4	28.1	31.0	23.6	23.6	31.3	28.8	34.4	33.9	0.008
		Among all last authors	20.4	23.6	26.3	15.5	10.9	18.2	20.8	27.3	21.9	33.9	0.253
Original articles													
	Female surgeon authors												
		Among all first authors of original articles	5.6	5.5	7.0	3.4	5.5	7.3	4.2	12.1	17.2	14.3	0.013
		Among all last authors of original articles	1.9	5.5	3.5	3.4	1.8	7.3	2.1	7.6	14.1	10.7	0.020

Representation of female surgeon authorship over the years

Regarding surgeon authors of original articles, we observed a trend of linear increase during the period analyzed in the proportion of female surgeons who were first authors (p<0.05) and last authors (p<0.05). The first surgeon authorship went from 5.6% in 2010 to 14.3% in 2019. The last surgeon authorship went from 1.9% in 2010 to 10.7% in 2019 (Table 2).

Authors who published the most

When ordering the authors by their number of publications during the analyzed period, we noticed that among the first 10 authors, one was a woman (ninth). The most published male author had his name appear three times more than the name of the most published female author. Of the 141 articles with these 10 authors, 10 had female authors (7.0%) (Table 3).

**Table 3:** Ranking of authors who published the most in the analyzed journal's editions *To hide the authors' real identity, we used fictional names

Order	Name*	Number of publications
1	James (man)	30
2	Robert (man)	16
3	John (man)	15
4	Michael (man)	15
5	William (man)	13
6	David (man)	12
7	Richard (man)	10
8	Joseph (man)	10
9	Mary (woman)	10
10	Charles (man)	10
	Total	141

## Discussion

To our knowledge, this is the first study to analyze the gender trends in the authorship of surgical literature in Brazil. We found a marked gender disparity in terms of the proportion of women and men authors in the analyzed journal, where only 27.8% were female. However, if we compare the data with the women's general prevalence in medicine (46.6% in 2020) [10] and surgery (22.1% general surgeons in 2020) [10] in Brazil, our study reveals an optimistic scenario of progress. Similarly, as in medicine and surgery fields in general, women's prevalence as manuscript authors has been markedly increasing over the years, as we noted in our study, rising from 22.2% in 2010 to 35.6% in 2019. Worldwide, women accounted for around 18.2-23.6% of the authors in surgical journals in 2003 [6].

Our research revealed a gender gap in the first and last author positions. Other studies have shown that identifying the female proportion in these authorship positions can help us gain an understanding of the advancement of women in their scientific careers [2]. The first author commonly plays the leadership role in the production of the paper while the last one holds a senior position. In our study, women represented only 25% of first authors and 21.8% of last authors. Among the original articles, they represented 27.3% of first authors and 22% of last authors. In Brazil, there have been a few studies assessing gender trends in the medical literature authorship. In a study of the Brazilian neurology journal, it was found that women represented 19.5% of the first authors and 18.4% of the senior authors [11]. Women are also underrepresented as first, senior, and editorial authors worldwide in many medical specialties such as internal medicine, obstetrics and gynecology, otorhinolaryngology, and ophthalmology [2,12,13].

Although the scenario is far from equal between men and women, there was a significant linear increase in the female representation among the first authors of original articles in recent years, from 20.4% in 2010 to 33.9% in 2019. Our result may reflect the growth of women's presence in the surgery specialty over the years. In 2011, 16.2% of all general surgeons in Brazil were female [14], and, in 2020, it rose to 21.1% [10]. Among the last authors, the same progress was not seen, with a decrease in 2013 (15.5%) and 2014 (10.9%). This scenario may reveal additional obstacles that women have to face to progress in their scientific careers and to achieve prestigious positions in academia. Despite the low representation, in our analysis of female surgeons, both as first and last authors, we noted a significant linear increase over the period considered. As we mentioned, this may be attributed to the increase in female surgeons in general in Brazil, and it can point to a slow progress, but a significant one for women in the surgical field.

The general gender authorship trends from a surgical journal provide valuable information about "who is publishing about surgery." It indirectly shows the literature produced by the specialists and published in that journal. However, it does not tell us much about the literature written by the surgeons themselves. With that in mind, we decided to investigate whether the authors were surgeons or not, in order to draw a more narrowed-down and clear picture. It involves an innovative analysis in the search for understanding the women surgeons' representation in the surgical literature authorship. In this context, we found that only 8.4% of first authors and only 6.1% of last authors of original articles were, in fact, women surgeons, while men comprised 52.5% of first and 53.3% of last authorship positions as surgeons. The contrasting data reveal a tremendous gender gap in these leadership positions in research, already described in the literature as the glass-ceiling phenomenon in surgical academia [15].

Among the 10 authors who published the most in the analyzed editions of the journal, there was only one woman, reaffirming the persistent male dominance within the academic environment. This female author contributed only 10 of the 141 articles in the ranking. The most frequent author participated in 30 articles. This leads us to the question: what are the factors that act as barriers to women publishing bigger volumes of research? Probably, this woman in the ranking is a senior researcher; however, she still published three times less than the most frequent author. This result may objectively indicate that women face far more challenges in scientific research than men.

The core objective in seeking an understanding of the gender gap in the authorship of surgical journals in Brazil is to generate a discussion on the topic and to encourage more investigations looking for the causes and consequences of this scenario. Studies show a multifactorial network of causes for such a disparity, including less financial support and research space available to female researchers compared to their male counterparts [16,17] and difficulty in finding mentors [18]. Gaining a deeper insight into the scenario can help us build compatible and effective solutions, such as mentoring programs [19,20], interventions that address subtle gender prejudices [21,22], and female-centric career development programs [23]. That way, not only the surgical field but also medicine, in general, can provide a level playing field for all future doctors, resulting in benefits for society as a whole.

Our study has several limitations. A major limitation is that we made an explicit decision to not reveal the name of the journal analyzed, due to ethical reasons related to research in Brazil. Moreover, due to a limitation inherent in an observational study such as this, we could not assess cause-consequence relations across the data observed. As the focus of our analysis was on surgeons, we are aware that we could have done more to explore the non-surgeon category, which would have contributed extra data to the literature. Additionally, we also decided not to include the middle authors; this decision was based on the fact that some articles did not have middle authors and also because this position has less scientific relevance compared to the first and last ones. The gender categorization based on names is another limitation since it may not correspond with the actual gender identity of the authors in some cases, even though the method is a widely adopted one in the literature. Another limitation is that our analysis was confined to only one surgical journal published out of Brazil, and therefore, despite the significant sample size, our results may not represent the entire country. Although our findings have clearly demonstrated the differences in publication output between men and women, we could not conclusively establish the fundamental aspects that lead to such a scenario.

## Conclusions

Women are still underrepresented in surgical literature authorship in Brazil. Their contribution is minuscule in general, as well as in the role of first and last authors. When the analysis is narrowed down to surgeon authors, in particular, the gap is enormous in terms of the representation of women surgeons. Despite the gaps described, our study showed that women's proportion in authorship has been increasing over the years. It represents an optimistic scenario for the next generation. We recommend further studies that address the possible causes and potential solutions to rectify this issue in the long run.

## References

[1] Colling Colling, A A (2020). Colling A: The first Brazilian doctors - women ahead of their time (Site in Portuguese). https://ojs.ufgd.edu.br/index.php/FRONTEIRAS/article/view/1607/964.

[2] Jagsi R, Guancial EA, Worobey CC (2006). The "gender gap" in authorship of academic medical literature--a 35-year perspective. N Engl J Med.

[3] Bickel J (1988). Women in medical education. A status report. N Engl J Med.

[4] Bickel J, Wara D, Atkinson BF (2002). Increasing women's leadership in academic medicine: report of the AAMC Project Implementation Committee. Acad Med.

[5] Nonnemaker L (2000). Women physicians in academic medicine: new insights from cohort studies. N Engl J Med.

[6] Kurichi JE, Kelz RR, Sonnad SS (2005). Women authors of surgical research. Arch Surg.

[7] Okike K, Liu B, Lin YB (2012). The orthopedic gender gap: trends in authorship and editorial board representation over the past 4 decades. Am J Orthop (Belle Mead NJ).

[8] (2020). Federal Medical Council of Brazil (database online). https://portal.cfm.org.br/index.php.

[9] (2020). Brazilian National Council for Scientific and Technological Development. http://buscatextual.cnpq.br/buscatextual/busca.do.

[10] Scheffer M, Cassenote A, Guerra A (2021). Medical demography in Brazil 2020 (Content in Portuguese). https://www.fm.usp.br/fmusp/conteudo/DemografiaMedica2020_9DEZ.pdf.

[11] Takayanagui OM, Livramento JA (2009). The increasing female participation in authorship of articles published in neurology in Brazil. Arq Neuropsiquiatr.

[12] Filardo G, da Graca B, Sass DM, Pollock BD, Smith EB, Martinez MA (2016). Trends and comparison of female first authorship in high impact medical journals: observational study (1994-2014). BMJ.

[13] Bhattacharyya N, Shapiro NL (2000). Increased female authorship in otolaryngology over the past three decades. Laryngoscope.

[14] Scheffer M, Biancarelli A, Cassenote A (2021). Medical demography in Brazil (Content in Portuguese). https://portal.cfm.org.br/images/stories/pdf/demografiamedicanobrasil.pdf.

[15] Zhuge Y, Kaufman J, Simeone DM, Chen H, Velazquez OC (2011). Is there still a glass ceiling for women in academic surgery?. Ann Surg.

[16] Cain JM, Schulkin J, Parisi V, Power ML, Holzman GB, Williams S (2001). Effects of perceptions and mentorship on pursuing a career in academic medicine in obstetrics and gynecology. Acad Med.

[17] Benz EJ Jr, Clayton CP, Costa ST (1998). Increasing academic internal medicine's investment in female faculty. Am J Med.

[18] Yedidia MJ, Bickel J (2001). Why aren't there more women leaders in academic medicine? The views of clinical department chairs. Acad Med.

[19] Bates C, Gordon L, Travis E (2016). Striving for gender equity in academic medicine careers: a call to action. Acad Med.

[20] Varkey P, Jatoi A, Williams A (2012). The positive impact of a facilitated peer mentoring program on academic skills of women faculty. BMC Med Educ.

[21] Westring A, McDonald JM, Carr P, Grisso JA (2016). An integrated framework for gender equity in academic medicine. Acad Med.

[22] Carnes M, Devine PG, Baier Manwell L (2015). The effect of an intervention to break the gender bias habit for faculty at one institution: a cluster randomized, controlled trial. Acad Med.

[23] Helitzer DL, Newbill SL, Cardinali G, Morahan PS, Chang S, Magrane D (2016). Narratives of participants in national career development programs for women in academic medicine: identifying the opportunities for strategic investment. J Womens Health (Larchmt).

